# The NK cell checkpoint NKG2A maintains expansion capacity of human NK cells

**DOI:** 10.1038/s41598-023-37779-6

**Published:** 2023-06-29

**Authors:** Meike Kaulfuss, Juliane Mietz, Astrid Fabri, Johannes vom Berg, Christian Münz, Obinna Chijioke

**Affiliations:** 1 Cellular Immunotherapy, Institute of Experimental Immunology, University of Zürich, Zurich, Switzerland; 2 Institute of Immunity & Transplantation, University College London Division of Infection & Immunity, London, UK; 3 Institute of Laboratory Animal Science, University of Zürich, Schlieren, Switzerland; 4 Viral Immunobiology, Institute of Experimental Immunology, University of Zürich, Zurich, Switzerland; 5 Institute of Medical Genetics and Pathology, University Hospital Basel, Basel, Switzerland

**Keywords:** Immunotherapy, Lymphocytes, NK cells

## Abstract

Human natural killer (NK) cells are cytotoxic effector cells that are increasingly harnessed in cancer immunotherapy. NKG2A/CD94 is an inhibitory receptor on NK cells that has established regulatory functions in the direct interaction with target cells when engaged with its ligand, the non-classical HLA class I molecule HLA-E. Here, we confirmed NKG2A as a checkpoint molecule in primary human NK cells and identified a novel role for NKG2A in maintaining NK cell expansion capacity by dampening both proliferative activity and excessive activation-induced cell death. Maintenance of NK cell expansion capacity might contribute to the preferential accumulation of human NKG2A^+^ NK cells after hematopoietic cell transplantation and enrichment of functionally impaired NK cells in human cancers. Functional silencing of NKG2A for cancer immunotherapy is highly attractive but will need to consider that this might also lead to a reduced survival by driving activation-induced cell death in targeted NK cells.

## Introduction

Human natural killer (NK) cells have received renewed attention in clinical immuno-oncology, especially for use in adoptive cell therapy^1,2^, most recently as engineered cytotoxic effector cells carrying chimeric antigen receptors for the treatment of hematologic cancers^3^. Advantages of targeting NK cells compared to T cells for cancer immunotherapy include their innate antitumor activity and, by demonstrating a remarkable safety profile^3,4^, their potential as off-the-shelf product. However, adoptive transfer of non-engineered NK cell products or singular targeting of inhibitory receptors on endogenous NK cells by means of antagonistic antibodies have not yet yielded substantial clinical benefit^2^. Therefore, a better understanding of NK cell biology seems critical to better harness and enhance the antitumor potential of these cytotoxic cells.

The surface molecule NKG2A complexed as a heterodimer with the non-signaling CD94 molecule is an invariant inhibitory NK cell receptor (hereafter referred to as NKG2A)^5–7^. NKG2A has established regulatory functions in the direct interaction of NK cells with target cells, limiting NK cell effector responses like cytotoxicity when engaged with its ligand, the non-classical HLA class I molecule HLA-E^5^. Importantly, overexpression of HLA-E has been reported in a wide range of human cancers^8^ and has been associated with worse prognosis in colorectal cancer, non-small cell lung carcinoma and gynecological cancers^9–13^. Blocking NKG2A or disruption of its ligand on cancer cells has been demonstrated to improve outcome of immunotherapy and thus, NKG2A has been considered an NK cell checkpoint^8,14–17^. Furthermore, NKG2A is involved in NK cell education^18,19^ rendering NKG2A^+^ NK cells more reactive towards transformed HLA class I negative target cells. Since in humans NKG2A is expressed by around half of blood NK cells^20^, NKG2A with its low genetic diversity makes it an abundant and attractive target molecule for engineering of NK cells.

Here, we disrupted NKG2A via CRISPR gene editing and expanded engineered NK cells, which is needed to reach large enough numbers for therapeutic infusions. We adopted an ex vivo NK cell expansion protocol that has been used safely in clinical trials^3,21^, based on a myeloid leukemic cell line expressing membrane bound IL-21 as irradiated feeder cells^22^. Although expression of NKG2A has been associated with an immature NK cell phenotype that correlates with increased proliferative capacity^23^, it remained unclear whether NKG2A is directly involved in NK cell expansion. By abrogating NKG2A at the genetic level, we validated the functional involvement of NKG2A in NK cell cytotoxicity and provide evidence suggesting that NKG2A is required to maintain NK cell expansion.

## Methods

### Primary cells and cell lines

Peripheral blood mononuclear cells (PBMCs) were isolated from healthy donors with density gradient centrifugation (Ficoll Paque Premium, GE Healthcare, 17-5442-03). For the expansion of NKG2A^+^ and NKG2A^−^ NK cells, PBMCs were first depleted of T cells with anti-CD3 MicroBeads (Miltenyi, 130-050-101) and in a second step separated by using APC-conjugated anti-CD159a (Miltenyi, 130-113-563) and anti-APC MicroBeads (Miltenyi, 130-090-855) according to the manufacturer’s instructions. Primary human NK cells were isolated from PBMCs using the human NK cell Isolation Kit (Miltenyi, 130-092-657) for negative selection of NK cells and the autoMACS^®^ Pro Separator according to manufacturer’s instructions. For expansion of NK cells, irradiated (130 Gy) K562mbIL21 feeder cells (kindly provided by Dr. Dean Lee, Nationwide Children’s Hospital, Columbus, United States) were added at a 1:2 ratio (lymphocyte to feeder cell ratio) on day 0 and at a 1:1 ratio every 7 days. Transformed autologous cells were manufactured from primary human B cells, isolated from PBMCs using anti-CD19 MicroBeads (Miltenyi, 130-050-301) according to the manufacturer’s protocol and infected with Epstein Barr Virus (EBV) B95-8 at an MOI of 0.1 for transformation, to generate lymphoblastoid cell lines (LCLs). Primary human NK cells were cultured in RPMI1640 medium (Gibco) supplemented with 10% fetal calf serum (FCS; Biochrom), 1% penicillin/streptomycin (1% P/S; Gibco) and 200 U/mL recombinant human IL-2 (PeproTech, 200-02). Medium was replaced every 2–3 days. K562 cells were obtained from ATCC. K562 cells, LCLs and K562mbIL21 cells were cultured in RPMI1640 medium supplemented with 10% FCS and 1% P/S and passaged twice per week. Viability of the cells was assessed using 0.4% trypan blue solution (Gibco) at a 1:10 dilution.

### Editing of NK cells

The NKG2A knockout (KO) from bulk NKG2A^+^ NK cells and NKG2A^+^KIR^−^ NK cells was performed 7 days after start of the expansion using CRISPR gene editing. The *KLRC1* targeting crRNA (ACTGCAGAGATGGATAACCA) was designed in-house with CRISPOR^24^ and purchased from Integrated DNA Technologies (IDT) as was the non-targeting control crRNA, the tracrRNA (IDT, 1072533) and Cas9 (*Streptococcus pyogenes*) protein (IDT, 1081058). The formation of Cas9 ribonucleoproteins (RNPs) was performed as described by Roth et al.^25^ 0.5 × 10^6^ NK cells were electroporated per well of a Nucleostrip™ with RNPs using the P3 Primary Cell 4D-Nucleofector™ X kit S (Lonza, V4XP-3032) according to the manufacturer’s instructions and pulsed with the code DK-100 using the 4D-Nucleofector™ (Lonza). NKG2A KO efficiency was determined 72 h after electroporation by flow cytometry.

### Flow cytometry

For the detection of surface markers cells were stained for 20 min at 4 °C in PBS with the appropriate antibodies. For the detection of intracellular or intranuclear markers after surface staining the cells were fixed with the BD Cytofix/Cytoperm™ Fixation/Permeabilization Kit (BD, 554714) according to the manufacturer’s instructions and stained with the appropriate antibodies for 30 min at 4 °C. For the detection of dead cells, the samples were stained with Zombie Aqua™ (Biolegend, 423101). Samples were acquired on BD LSRFortessa™ Flow Cytometer (BD Biosciences) or BD FACSymphony 5L (BD Biosciences). To evaluate apoptosis, cells were stained with the surface markers first, then washed and stained with Annexin V (IQ products, IQP-120F) according to the manufacturer’s instructions. Cells were defined as apoptotic when being Annexin V positive and negative for the live/dead marker. The data was analyzed using FlowJo software (Tree Star). The following antibodies were used: anti-CD56 (BV650, HCD56, Biolegend, 318344), anti-CD16 (BV605, 3G8, Biolegend, 302040), anti-CD3 (BV711, OKT3, Biolegend, 317328), anti-CD3 (PerCP-Cy5.5, UCHT1, Biolegend, 300430), anti-Ki67 (BV605, 16A8, Biolegend, 652413), anti-CD57 (PE-Dazzle-594, HNK1, Biolegend, 359620), anti-CD159a (FITC, REA110, Miltenyi, 130-113-565), anti-phosphorylated S6 ribosomal protein (PE-Cy7, Cell signaling, 34411S), anti-CD25 (APC-Cy7, BC96, Biolegend, 302614), anti-CD132 (PE, TUGh4, Biolegend, 338606), anti-CD158a,h (APC, EB6B, Beckmann Coulter, A22332), anti-CD158b1/b2,j (APC, GL183, Beckmann Coulter, A22333), anti-CD158e (APC, DX9, R&D, FAB1225A-100), anti-CD159a (PE, Z199, Beckmann Coulter, IM2391U), anti-LILRB1 (PE-Cy7, HP-F1, Invitrogen/eBioscience, 25-5129-42), anti-phospho-STAT5-Y694 (PerCP-eFluor710, SRBCZX, Invitrogen/eBioscience, 46-9010-42), anti-phospho-STAT3-Y705 (PE-Cy7, LUVNKLA, Invitrogen/eBioscience, 25-9033-42), anti-HLA-ABC (APC-Cy7, w6/32, Biolegend, 311426), anti-HLA-E (APC, 3D12, Biolegend, 342606). For staining of pSTAT3 and pSTAT5, cells were fixed with the BD Pharmingen Transcription Factor Phospho Buffer Set (BD, 565575) following the manufacturer’s instructions.

### Cytotoxicity assays

For cytotoxicity assays K562 cells or LCLs were labeled with PKH67 green (Sigma, MINI67-1KT) or PKH26 red fluorescent cell linker (Sigma, MINI26-1KT) according to the manufacturer’s protocol. Labeled target cells and primary cells were cocultured at different target:effector ratios (1:1, 1:5, 1:10, 1:20) for 4 h. Viability of the labeled target cells was determined by the addition of TO-PRO™-3 iodide (Invitrogen, T3605) immediately before acquisition.

### FACS sort

NK cells were stained for CD3, CD56, NKG2A, KIR2DL1/DS1, KIR2DL2/L3/S2, KIR3DL1 and live/dead as described above. After selection for viable CD3^−^CD56^+^ NK cells, subpopulations were sorted based on the expression of NKG2A (CD159a) and/or KIRs (KIR2DL1/DS1 (CD158a,h), KIR2DL2/L3/S2 (CD158b1/b2,j), KIR3DL1 (CD158e)) using a FACSAria III cell sorter with BD FACS Diva Software (BD Biosciences), nozzle size 100 µm. Purity of the populations was confirmed after sorting by flow cytometric analysis.

### Statistical analysis

Statistical analysis was performed with GraphPad Prism Version 9.2.0 (283). Number of experimental repeats, donors and specific statistical tests are specified in the corresponding figure legends. p values of less than 0.05 were considered significant.

### Ethics statement

All methods were carried out in accordance with relevant guidelines and regulations. Informed consent was obtained from all subjects. The use of blood from adult healthy donors was approved by and performed according to the cantonal ethical committee of Zürich, Switzerland (protocols KEK-StV-Nr. 19/08 and 2019-00837).

## Results

### Knockout of the NK cell checkpoint NKG2A increases killing of transformed autologous cells

**Figure 1:**
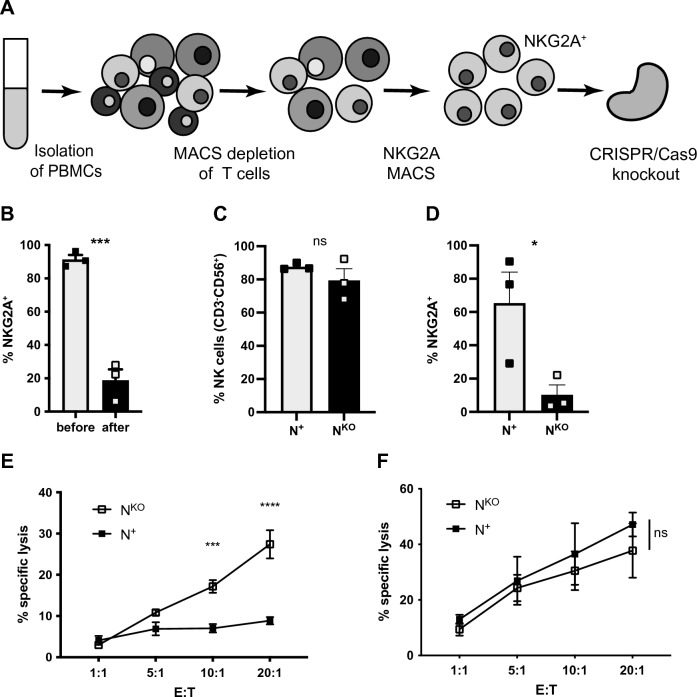
Knockout of the NK cell checkpoint NKG2A increases NK cell killing of transformed autologous cells. (**A**) PBMCs were isolated from healthy donors, depleted of T cells and selected for NKG2A^+^ cells by MACS. NKG2A was knocked out by nucleofection with Cas9 RNP complexes in enriched NKG2A^+^ cells. (**B**) Frequency of NKG2A before and after knockout (KO). (**C**) Frequency of CD3^−^CD56^+^ NK cells and (**D**) NKG2A expression in expanded NKG2A^+^ and NKG2A^KO^ subpopulations. (**E**) Specific lysis of autologous cells (Epstein Barr virus transformed B lymphoblastoid cells) or (**F**) of K562 cells by expanded NKG2A^+^ or NKG2A^KO^ NK cells at the indicated effector to target cell ratios (2way ANOVA, uncorrected Fisher’s LSD; shown is data from 2 independent experiments with 2 donors and 2 technical replicates each). N^+^, NKG2A^+^ NK cells; N^KO^, NKG2A^KO^ NK cells. Mean values ± SEM are shown, symbols represent single donors. Data in (**B**–**D**) includes 3 donors, significance by unpaired *t*-test. (**B**) ***p = 0.0005, (**D**) *p = 0.0476, (**E**) ***p = 0.0001, ****p < 0.0001.

To determine whether the cytotoxic capacity of human NK cells (Fig. S1 for gating strategy) could be increased by knocking out the inhibitory receptor NKG2A, PBMCs were isolated from healthy donors, depleted of T cells by magnetic-activated cell sorting (MACS), then separated by the expression of NKG2A and the NKG2A^+^ fraction selected (Fig. 1A). A crRNA targeting *KLRC1*, the gene encoding NKG2A located on chromosome 12 was designed in-house (Fig. S2A). Nucleofection of NKG2A^+^ cells with Cas9 ribonucleoprotein (RNP) complexes led to an average reduction of NKG2A protein expression by 78% as assessed by surface staining (Fig. 1B and Fig. S2B). Before performing cytotoxicity assays, no significant difference was found in the proportion of NK cells among NKG2A^+^ and NKG2A^KO^ populations (Fig. 1C) and NKG2A was still significantly reduced on NKG2A^KO^ NK cells after expansion (Fig. 1D and Fig. S2C). To determine the functional impact of the NKG2A knockout on NK cell cytotoxicity, expanded NKG2A^+^ or NKG2A^KO^ NK cells were co-cultured for 4 h with HLA class I positive autologous Epstein Barr virus transformed B lymphoblastoid cells at different effector to target cell ratios. Addition of NKG2A^KO^ NK cells resulted in significantly greater lysis of target cells than observed with addition NKG2A^+^ NK cells (Fig. 1E). In contrast, this increased killing capacity of NKG2A^KO^ NK cells compared to NKG2A^+^ NK cells was not detectable against K562 cells, a myelogenous leukemic cell line that is mostly HLA class I deficient and thus lacks the ligand for NKG2A (Fig. 1F). Together, these results demonstrate at the genetic level that NKG2A functions as a checkpoint molecule in human NK cells with disruption of *KLRC1* leading to increased cytotoxicity of engineered NKG2A^KO^ NK cells.

### NKG2A^−^ NK cells show a decreased fold expansion in vitro as compared to NKG2A^+^ NK cells

**Figure 2:**
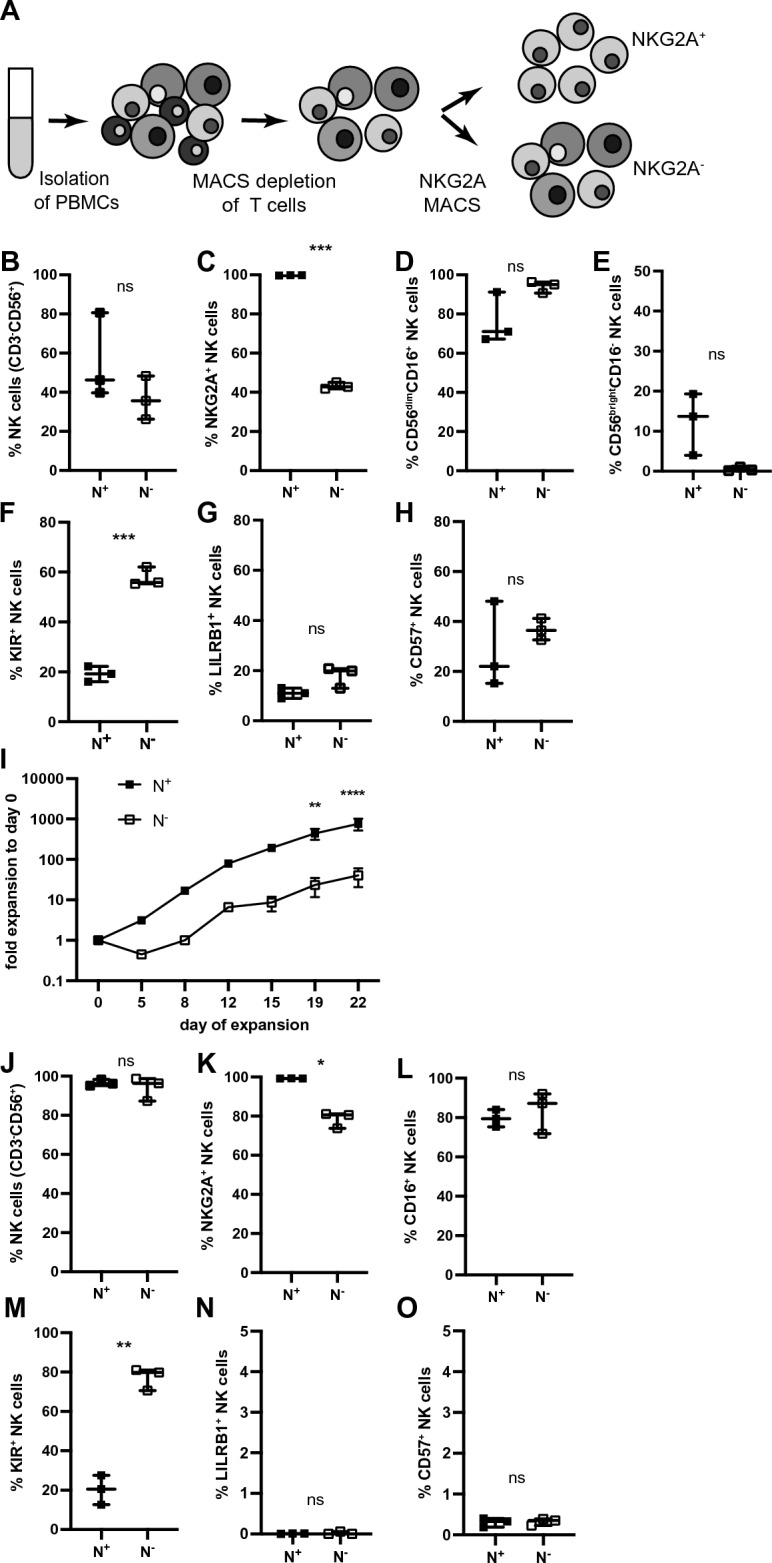
NKG2A^−^ NK cells show decreased expansion in vitro as compared to NKG2A^+^ NK cells. (**A**) PBMCs were isolated from healthy donors, depleted of T cells and separated into NKG2A^+^ and NKG2A^−^ cells by MACS. (**B**) The frequency of CD3^−^ CD56^+^ NK cells in both bulk subpopulations and (**C**) NKG2A expression within NK cells of both subpopulations after sort was determined by flow cytometry (paired *t*-test, ***p = 0.0003). (**D**–**H**) The frequency within NK cells in both subpopulations of CD56^dim^CD16^+^ cells (**D**), CD56^bright^CD16^−^ cells (**E**), expression of KIR (**F**, paired *t*-test ***p = 0.0006), LIRB1 (**G**) and CD57 (**H**) after sort was determined by flow cytometry. (**I**) Sorted NKG2A^+^ and NKG2A^−^ subpopulations were expanded for 21 days, adding irradiated K562mbIL21 feeder cells on day 0, 7 and 14 (2way ANOVA, Bonferroni’s multiple comparison, **p = 0.0040, ****p < 0.0001). (**J**) Frequency of CD3^−^CD56^+^ NK cells in sorted bulk subpopulations at the end of expansion (day 22). (**K**–**O**) Pre-gated on NK cells, expression of NKG2A (**K**, paired *t*-test, *p = 0.0130), CD16 (**L**), KIR (**M**, paired *t*-test **p = 0.0018), LILRB1 (**N**) and CD57 (**O**) at the end of expansion (day 22). N^+^, NKG2A^+^ NK cells; N^−^, NKG2A^−^ NK cells. Mean values ± min. and max. are shown, in (**I**) mean values ± SEM are shown. Symbols represent single donors; 3 donors.

In separate sets of experiments, NKG2A^−^ NK cells were isolated in parallel to NKG2A^+^ NK cells (Fig. 2A). After selection by MACS, frequencies of CD3^−^CD56^+^ NK cells were not significantly different between the NKG2A^+^ subpopulation and the NKG2A^−^ subpopulation (Fig. 2B). NKG2A expression on CD3^−^CD56^+^ NK cells was > 97% in the NKG2A^+^ subpopulation and below 50% within NK cells of the NKG2A^−^ subpopulation (Fig. 2C). Furthermore, the percentages of CD56^dim^CD16^+^ NK cells among sorted NKG2A^+^ NK cells and NKG2A^−^ NK cells (Fig. 2D) as well as the percentages of CD56^bright^CD16^−^ NK cells did not differ significantly between the two NK cell fractions, although CD56^bright^CD16^−^ NK cells were rare among NKG2A^−^ NK cells (Fig. 2E). In contrast, the proportion of cells with KIR expression was almost threefold higher in NKG2A^−^ NK cells than in NKG2A^+^ NK cells (Fig. 2F), while there was no significant difference in the expression of the inhibitory receptor LILRB1 (Fig. 2G) or the terminal differentiation marker CD57 (Fig. 2H). Compared to NKG2A^+^ NK cells, NKG2A^−^ NK cells showed a significantly lower fold bulk expansion over the course of 3 weeks (Fig. 2I, mean difference on day 22: 18.8-fold). Because of low NK cell frequencies in the starting population after MACS enrichment, NK cells were FACS sorted to high purity. Notably, the difference in expansion capacity was maintained in FACS sorted NK cells comparing NK cells after knocking out NKG2A via Cas9 RNPs and NKG2A^+^ NK cells treated with nontargeting Cas9 RNPs (Fig. S2D). At the end of expansion, both NKG2A^+^ and NKG2A^−^ MACS enriched subpopulations contained almost exclusively CD3^−^CD56^+^ NK cells (Fig. 2J). However, the percentage of NKG2A expression increased to 80% in the NKG2A^−^ subpopulation, whereas it remained close to 100% in the NKG2A^+^ subpopulation (Fig. 2K). While discrimination between CD56^dim^CD16^+^ NK cells and CD56^bright^CD16^−^ NK cells was no longer possible due to upregulation of CD56 during expansion, the percentage of CD16^+^ NK cells was similar in both NKG2A^+^ and NKG2A^−^ NK cell fractions (Fig. 2L). In contrast, KIR expression in NKG2A^+^ and NKG2A^−^ NK cells showed significant differences after expansion, which was higher in NKG2A^−^ NK cells (Fig. 2M), similar to the differences at baseline (Fig. 2F). Interestingly, the percentage LILRB1^+^ NK cells and CD57^+^ NK cells decreased to below 1% in both NKG2A^+^ and NKG2A^−^ NK cell fractions after expansion (Fig. 2N,O). Thus, NKG2A^−^ NK cells display reduced expansion capacity when stimulated with IL-2 and irradiated myeloid leukemic K562 feeder cells with membrane-bound IL-21 and maintain high KIR expression.

### NKG2A^−^ NK cells lacking KIR expression exhibit the lowest expansion capacity

**Figure 3:**
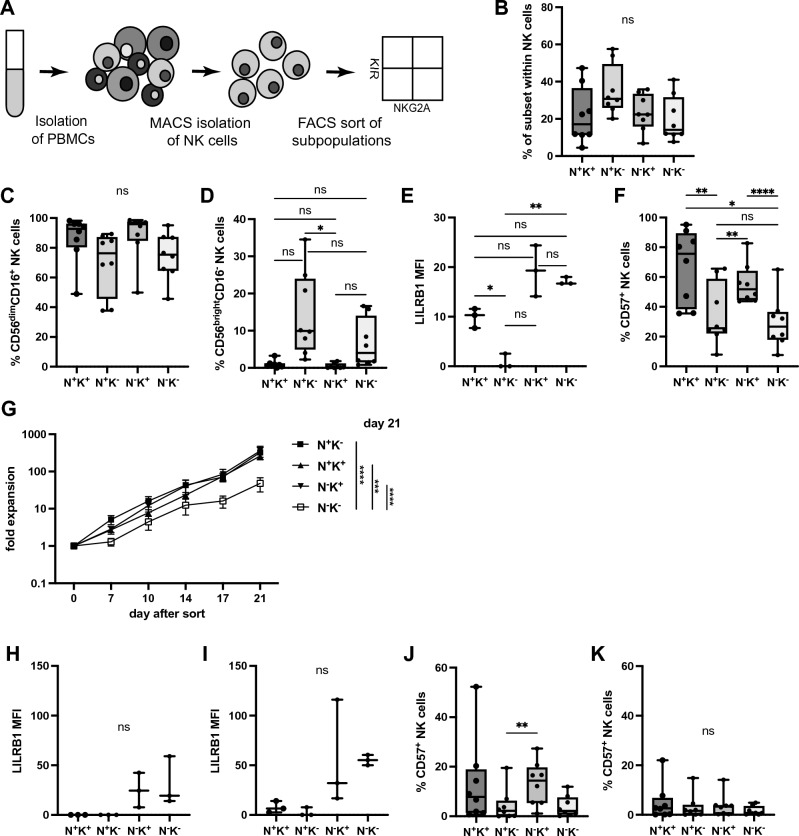
KIR expression rescues the expansion capacity of NKG2A^−^ NK cells. (**A**) PBMCs were collected from healthy donors, NK cells isolated by negative selection (MACS) and sorted by FACS into four NK cell subsets: NKG2A^+^KIR^+^, NKG2A^+^KIR^−^, NKG2A^−^KIR^+^, NKG2A^−^KIR^−^. (**B**) Proportion of the four NK cell subsets among bulk CD3^−^CD56^+^ NK cells before sort was determined by flow cytometry. (**C**–**F**) Frequency within sorted NK cell subsets of CD56^dim^CD16^+^ cells (**C**), CD56^bright^CD16^−^ cells (**D**, *p = 0.0499 for N^+^K^−^ vs N^−^K^+^), intensity of LIRB1 (**E**, *p = 0.0412 for N^+^K^+^ vs N^+^K^−^ ; **p = 0.0041 for N^+^K^−^ vs N^−^K^−^) and expression of CD57 (**F**, **p = 0.0022 for N^+^K^+^ vs N^+^K^−^ ; **p = 0.0068 for N^+^K^−^ vs N^−^K^+^; *p = 0.0114 for N^+^K^+^ vs N^−^K^−^; ****p < 0.0001 for N^−^K^+^ vs NK^−^) before start of the expansion by flow cytometry. (**G**) The four NK cell subsets were expanded for 21 days, adding irradiated K562mbIL21 feeder cells on day 0, 7 and 14 showing expansion of bulk populations from sorted subsets (***p = 0.0005 for N^+^K^+^ vs N^−^K^−^; ****p < 0.0001 for N^+^K^−^ vs N^−^K^−^; ****p < 0.0001 for N^−^K^+^ vs N^−^K^−^). (**H**,**I**) Intensity of LILRB on NK cell subsets on day 13 (**H**) and on day 20 (**I**) after the sort. (J, K) Expression of CD57 on NK cell subsets on day 13 (**J**, **p = 0.0076 for N^+^K^−^ vs N^−^K^+^) and on day 20 (**K**) after the sort. N^+^K^+^, NKG2A^+^KIR^+^ NK cells; N^+^K^−^, NKG2A^+^KIR^−^ NK cells; N^−^K^+^, NKG2A^−^KIR^+^ NK cells; N^−^K^−^, NKG2A^−^KIR^−^ NK cells. Mean values ± SEM are shown, symbols represent single donors; 3–8 donors. *p* values were calculated using 1way ANOVA with Bonferroni’s multiple comparison (**B**–**F**, **H**–**K**) and 2way ANOVA with Bonferroni’s multiple comparison (**G**).

Indeed, aside from NKG2A, killer cell immunoglobulin-like receptors (KIRs) are main regulators of NK cell responsiveness including NK cell education and, like NKG2A interact with HLA class I molecules^26^. Importantly, expression of both HLA-E and HLA class I on feeder cells is strongly increased upon coculture with NK cells (Fig. S3). Since expanded NKG2A^−^ NK cells upregulated NKG2A (Fig. 2K) but retained KIR expression (Fig. 2M) and to explore whether NK cell expansion is influenced by NKG2A depending on KIRs, NK cells were MACS isolated from PBMCs by negative selection, and four different subpopulations based on the expression of NKG2A and KIRs were sorted using flow cytometry: NKG2A^+^KIR^−^, NKG2A^+^KIR^+^, NKG2A^−^KIR^+^, NKG2A^−^KIR^−^ (Fig. 3A and Fig. S4A). The proportion of the different subsets varied among individuals, but the frequencies of the individual subpopulations were not significantly different across donors (Fig. 3B). There was no obvious difference in the proportion of CD56^dim^CD16^+^ NK cells within the four subsets (Fig. 3C). However, whereas the NKG2A^+^KIR^−^ cell subset contained more CD56^bright^CD16^−^ NK cells than the NKG2A^−^KIR^+^ cell subset, of note, by comparison, CD56^bright^CD16^−^ NK cells were at least equally abundant in the NKG2A^−^KIR^−^ cell subset and were not depleted (Fig. 3D). The intensity of LILRB1 was higher in NK cell subsets lacking NKG2A (Fig. 3E). Notably, CD57^+^ NK cells were not enriched in the NKG2A^−^KIR^−^ cell subset (Fig. 3F). The four subsets were then expanded separately over a course of 21 days. Compared to all other subsets, the double negative NKG2A^−^KIR^−^ NK cell subset showed the lowest capacity of bulk expansion (Fig. 3G) with log-fold differences at day 21 compared to the NKG2A^+^KIR^−^ subset (mean difference: 276.7-fold), the NKG2A^+^KIR^+^ subset (mean difference: 211.1-fold) and the NKG2A^−^KIR^+^ subset (mean difference: 311.5-fold). Likewise, expansion capacity with fold-expansion corresponding to initially sorted phenotype showed a similar deficit in the NKG2A^−^KIR^−^ NK cell subset (Fig. S5A). No significant differences in LILRB1 intensity at day 13 (Fig. 3H) or day 20 (Fig. 3I) were observed between expanding NK cell subsets after sort, although intensity tended to be higher in NK cell subsets lacking NKG2A. Together, since expansion capacity of sorted NK cell subsets lacking NKG2A differed according to expression of KIR and NK cell subsets with NKG2A expression maintained robust expansion, these data suggest a role for NKG2A and KIR in sustaining expansion capacity of NK cells.

### High frequency of CD57 expression does not mark NK cell subsets with diminished expansion capacity

CD57 is an established marker of a terminally differentiated subset of NK cells thought to harbor decreased proliferative potential^23,27,28^, however, heterogeneity within this subset has recently been described^29^. Expression of CD57 at steady state before the start of the expansion was highest on NKG2A^+^KIR^+^ NK cells and lower on double negative NKG2A^−^KIR^−^ NK cells compared to NKG2A^−^KIR^+^ NK cells (Fig. 3F and Fig. S4B). However, on day 13 (Fig. 3J and Fig. S4C) and day 20 (Fig. 3K and Fig. S4D) of expansion after the sort, CD57 expression within populations gradually decreased in all subsets with no significant differences between the individual subsets. Thus, even though the terminal differentiation marker CD57 was initially expressed at equal or higher frequencies (highest on NKG2A^−^KIR^+^ NK cells that showed the greatest fold expansion, Fig. 3G), all of these NK cell subsets displayed log-fold higher expansion capacity compared to the NKG2A^−^KIR^−^ NK cell subset that initially contained a majority of CD57^−^ cells. Therefore, at least in the context of the expansion protocol used herein, expression of CD57 per se seems not to predict proliferative potential of a given NK cell population.

### Expansion capacity of NKG2A^+^KIR^−^ NK cells directly depends on NKG2A

**Figure 4:**
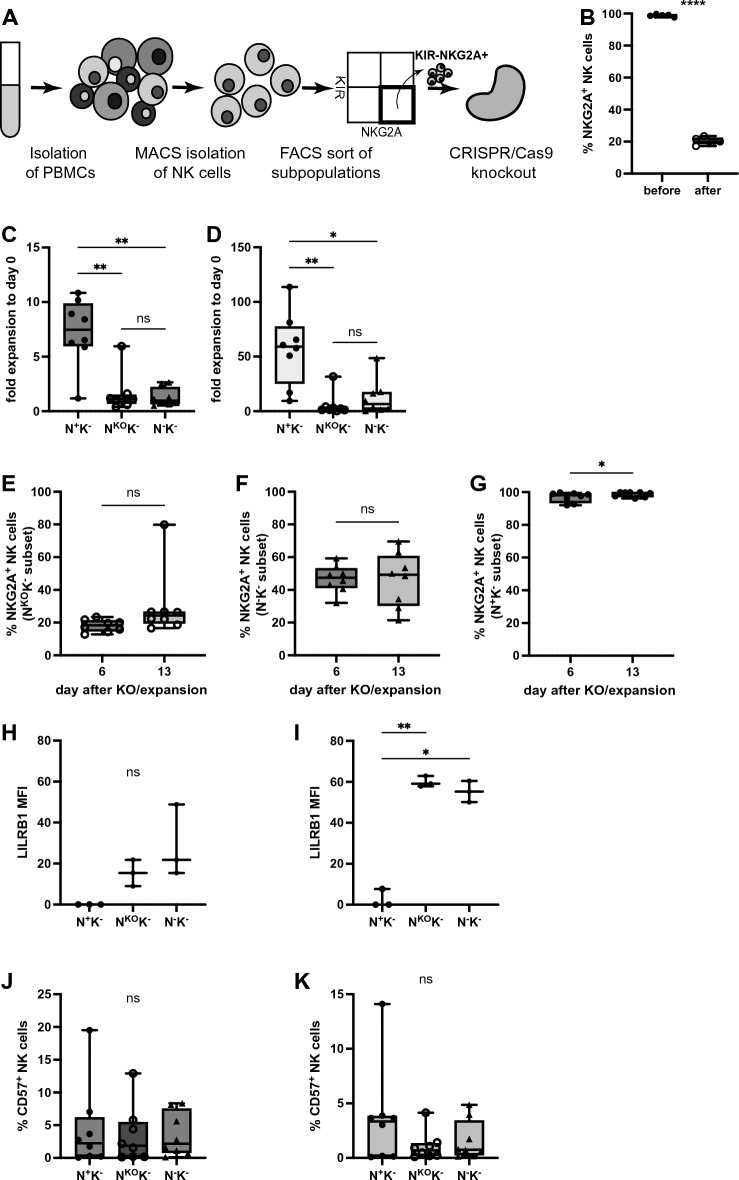
Cas9 RNP mediated NKG2A knockout in NKG2A^+^KIR^−^ NK cells leads to diminished expansion capacity. (**A**) NK cells were isolated from PBMCs by negative selection (MACS) and NKG2A^+^KIR^−^ NK cells and NKG2A^−^KIR^−^ NK cells sorted by FACS. NKG2A was knocked out by nucleofection with Cas9 RNP complexes in sorted NKG2A^+^KIR^−^ NK cells. (**B**) Surface expression of NKG2A on NKG2A^+^KIR^−^ NK cells before and after knockout (paired *t*-test, ****p < 0.0001). (**C**,**D**) NKG2A^+^KIR^−^ NK cells, NKG2A^KO^KIR^−^ and NKG2A^−^KIR^−^ NK cells were expanded for 21 days, adding irradiated K562mbIL21 feeder cells on day 0, 7 and 14. Expansion of bulk populations from sorted subsets at day 7 (**C**, **p = 0.0056 for N^+^K^−^ vs N^KO^K^−^; **p = 0.0015 for N^+^K^−^ vs N^−^K^−^ ) and day 14 (**D**, **p = 0.0059 for N^+^K^−^ vs N^KO^K^−^; *p = 0.0275 for N^+^K^−^ vs N^−^K^−^) after knockout. (**E**–**G**) Expression of NKG2A on day 6 and 13 after knockout in bulk populations of NKG2A^KO^KIR^−^ NK cells (**E**), NKG2A^−^KIR^−^ NK cells (**F**) and NKG2A^+^KIRNK cells (**G**, paired *t*-test, *p = 0.0153). (**H**,**I**) Intensity of LILRB1 on NK cell subsets on day 6 (**H**) and on day 13 (**I**, **p = 0.006 for N^+^K^−^ vs N^KO^K^−^; *p = 0.0185 for N^+^K^−^ vs N^−^K^−^) after knockout. (**J**,**K**) Expression of CD57 on NK cell subsets on day 6 (**J**) and on day 13 (**K**) after knockout. N^+^K^−^, NKG2A^+^KIR^−^ NK cells; N^KO^K^−^, NKG2A^KO^KIR^−^ NK cells; NKG2A^−^KIR^−^ NK cells. Mean values ± SEM are shown, symbols represent single donors; 3–8 donors. *p* values were calculated using paired *t*-test (**B**,**E**–**G**) and 1way ANOVA with Bonferroni’s multiple comparison (**C**,**D**,**H**–**K**).

To further investigate whether NKG2A is only a surrogate marker for more immature NK cells that possess increased proliferative potential or plays a direct role in KIR-independent expansion capacity of NK cells and because NKG2A^−^KIR^−^ NK cells had the lowest expansion (Fig. 3G), NKG2A was knocked out using Cas9 RNPs in sorted NKG2A^+^KIR^−^ NK cells (Fig. 4A), a subset that otherwise exhibits high expansion capacity (Fig. 3G). After the knockout, expression of NKG2A was efficiently abrogated in NKG2A^KO^KIR^−^ NK cells (Fig. 4B). When comparing the expansion capacity to parental NKG2A^+^KIR^−^ NK cells, the fold expansion of the NKG2A^KO^KIR^−^ population was significantly lower than that of NKG2A^+^KIR^−^ NK cells by day 7 (Fig. 4C, mean difference = 5.6) and day 14 (Fig. 4D, mean difference = 51.3) after knockout, closely resembling reduced expansion of NKG2A^−^KIR^−^ NK cells. Expansion capacity in NKG2A^KO^KIR^−^ and NKG2A^−^KIR^−^ NK cells with fold expansion corresponding to engineered or initially sorted phenotype also showed similar deficits (Fig. S5B,C). NKG2A expression remained at reduced levels in NKG2A^KO^KIR^−^ NK cells (Fig. 4E) but increased in NKG2A^−^KIR^−^ NK cells (Fig. 4F), while in the NKG2A^+^KIR^−^ population it was high and even increased slightly over the course of the expansion (Fig. 4G). Intensity of LILRB1 was not different on day 6 (Fig. 4H and Fig. S6A), but higher in both expanding NKG2A^KO^KIR^−^ and NKG2A^−^KIR^−^ NK cells on day 13 after knockout compared to NKG2A^+^KIR^−^ NK cells (Fig. 4I) and mock edited NKG2A^+^KIR^−^ NK cells (Fig. S6B). No significant differences were found in CD57 expression between expanding NKG2A^+^KIR^−^, NKG2A^KO^KIR^−^ and NKG2A^−^KIR^−^ NK cells on day 6 (Fig. 4J) and day 13 (Fig. 4K) after knockout or expanding mock edited NKG2A^+^KIR^−^ NK cells (Fig. S6C,D). Taken together, these results suggest a direct and KIR-independent role for the inhibitory receptor NKG2A in maintaining the expansion capacity of NK cells.

### Proliferation of NKG2A^KO^KIR^−^ NK cells cannot compensate for higher apoptosis rate

To assess whether a difference in the expression of receptors for interleukins that are associated with the expansion of NK cells could be involved in the reduced expansion capacity of NKG2A^KO^KIR^−^ NK cells, we investigated expression of unique and common interleukin receptor subunits by flow cytometry. An important interleukin for the activation and expansion of NK cells is interleukin-2 (IL-2), which we used in the expansion protocol alongside stimulation with membrane-bound IL-21^3,21,22^. However, only low levels of the IL-2 receptor alpha chain (CD25) could be detected with no significant differences between expanding NKG2A^+^KIR^−^ NK cells and NKG2A^KO^KIR^−^ NK cells on day 6 (Fig. S7A) and day 13 (Fig. S7B) after knockout, but trending higher in NKG2A^KO^KIR^−^ NK cells. Similarly, on day 6, no significant differences were observed for the expression of the common cytokine receptor gamma chain (CD132) which is the receptor subunit for multiple interleukins including IL-2, IL-15 and IL-21 (Fig. S7C). However, there was a slight decrease in CD132 intensity on expanding NKG2A^KO^KIR^−^ NK cells by day 13 after knockout (Fig. S7D). Staining for the IL-21 receptor did not reveal distinct expression in NKG2A^+^KIR^−^ NK cells from NKG2A^KO^KIR^−^ NK cells (data not shown). To explore signaling downstream of receptor engagement upon IL-2 binding, phosphorylation of STAT5 was compared in expanding NKG2A^+^KIR^−^ NK cells and NKG2A^KO^KIR^−^ NK cells, showing a slight decrease in NKG2A^KO^KIR^−^ NK cells at day 6 after knockout (Fig. S7E), but no differences at day 13 (Fig. S7F). Likewise, as a readout for signaling downstream of receptor engagement upon IL-21 binding, phosphorylation of STAT3 did not reveal any obvious differences in expanding NKG2A^+^KIR^−^ NK cells and NKG2A^KO^KIR^−^ NK cells (Fig. S7G,H). Therefore, these data indicate that differential expression of receptors for interleukins and associated downstream signaling relevant to NK cell activation and survival is not critically involved in the different expansion capacity of NKG2A^+^KIR^−^ NK cells versus NKG2A^KO^KIR^−^ cells.

The mTOR signaling pathway directs cellular growth and maintains cellular homeostasis^30^. The phosphorylated ribosomal protein S6 (p-S6) is a downstream target of mTORC1 and thus serves as a marker of mTORC1 activity^31,32^. To assess mTORC1 activity, expression of p-S6 was measured, but p-S6 was not differentially expressed in NKG2A^+^KIR^−^ NK cells compared to NKG2A^KO^KIR^−^ cells neither on day 6 (Fig. S7I) nor day 13 (Fig. S7J) of expansion. These data suggest that differences in mTORC1 activity are not the primary cause of the reduced expansion capacity of NKG2A^KO^KIR^−^ cells.

**Figure 5:**
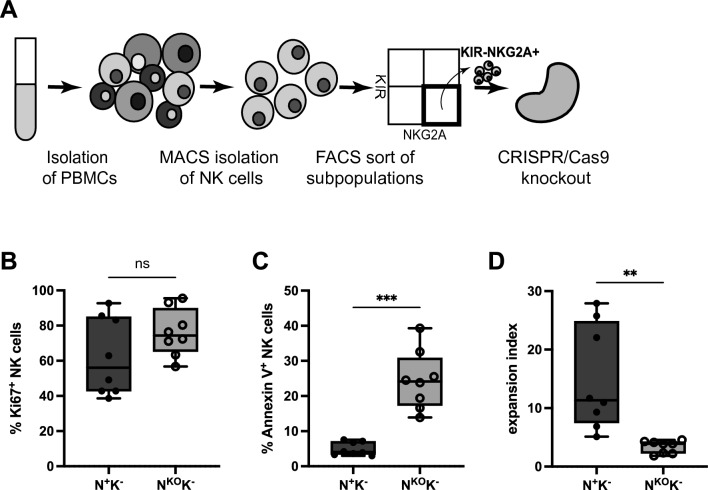
Proliferation of NKG2A^KO^ NK cells cannot compensate for higher apoptosis rate. (**A**) NK cells were isolated from PBMCs by MACS negative selection and NKG2A^+^KIR^−^ NK cells sorted by FACS. NKG2A was knocked out by nucleofection with Cas9 RNP complexes in sorted NKG2A^+^KIR^−^ NK cells. Both NKG2A^+^KIR^−^ and NKG2A^KO^KIR^−^ NK cells were expanded with K562mbIL21 feeder cells. (**B**) Ki-67 as a marker for proliferation was measured by flow cytometry on day 6 after knockout in NKG2A^KO^KIR^−^ and NKG2A^+^KIR^−^ NK cells. (**C**) Annexin V binding as a marker for apoptosis was measured by flow cytometry on day 6 after knockout in NKG2A^KO^KIRand NKG2A^+^KIR^−^ NK cells (***p = 0.0004). (**D**) The ratio of Ki-67 expression to Annexin V binding (expansion index) on day 6 after knockout in NKG2A^KO^KIR^−^ and NKG2A^+^KIR^−^ NK cells (**p = 0.0097). N^+^K^−^, NKG2A^+^KIR^−^ NK cells; N^KO^K^−^, NKG2A^KO^KIRNK cells. Mean values ± SEM are shown, symbols represent single donors; 8 donors. *p* values were calculated using paired *t*-test.

When measuring proliferation and apoptosis after NKG2A knockout (Fig. 5A), the expression of the proliferation marker Ki-67 in NKG2A^KO^KIR^−^ NK cells trended to be increased but not significantly higher than in NKG2A^+^KIR^−^ cells NK on day 6 after knockout (Fig. 5B). Apoptotic cells were detected using Annexin V^33^ (Fig. S8A), and interestingly NKG2A^KO^KIR^−^ NK cells showed a more than three-fold higher frequency of Annexin V binding as compared to NKG2A^+^KIR^−^ NK cells (Fig. 5C). However, when comparing the ratio of proliferation to apoptosis, the proliferation rate of NKG2A^KO^KIR^−^ NK cells could not compensate for the drastically higher apoptosis and thus the expansion index, i.e., the Ki-67^+^/Annexin V^+^ ratio, was significantly decreased for NKG2A^KO^KIR^−^ NK cells (Fig. 5D and Fig. S8D). These results indicate that NKG2A buffers both proliferative activity and excessive activation-induced cell death to maintain expansion capacity of human NK cells.

## Discussion

Our data demonstrate that primary human NK cells can be gene edited to efficiently abrogate NKG2A protein expression and confirm at the genetic level that NKG2A functions as an NK cell checkpoint molecule. A function of NKG2A in NK cell biology beyond its role in inhibiting immediate effector function and mediating NK cell education, however, has remained largely unexplored. Recently, NKG2A has been demonstrated to inhibit short-term NK cell proliferation^34^. In contrast, we here show that during expansion over a period of several weeks, NKG2A is essential for maintaining the expansion capacity of human NK cells. Our data suggest that NKG2A dependent expansion is achieved through regulation of proliferative activity and apoptosis to avoid activation-induced cell death. The results by Anton et al.^34^ and our current work do not need to be mutually exclusive, however, since the observed inhibition of proliferative activity is likely to precede the impact of activation-induced cell death on expansion. Thus, the NKG2A dependent reduction in proliferative activity in the initial phase of expansion described by Anton et al. may initially mask the increased expansion capacity we observed, which is also regulated by NKG2A but only becomes apparent later during expansion. We did not identify a major contribution of differences in IL-2 or IL-21 signal integration mediated by NKG2A in regulating NK cell expansion capacity. While other work has described downregulation of the IL-2 receptor subunit CD25 after abrogation of NKG2A surface expression^8^, we, like others^35^, only found low expression of CD25 without evidence of further decrease after genetic disruption of NKG2A. Furthermore, we found that the frequency of CD57 expression, a marker of terminal NK cell differentiation^23,27,28^, was unable to predict expansion capacity and decreased in expanding NKG2A^+^ NK cells. These data suggest that NKG2A can shield NK cells from apoptotic activation-induced cell death and terminal differentiation, leading to the depletion of CD57^+^ NK cells.

The myeloid K562 cells used in the expansion protocol may partially reflect the physiological interaction of NK cells and myeloid cells, which is particularly relevant in the tumor microenvironment^36,37^, providing signals like cytokines and ligands, e.g., the NKp30 ligand for NK cell activation^38–40^. Interestingly, reconstitution of NK cells in patients after hematopoietic cell transplantation (HCT) is dominated by NKG2A^+^ NK cells with reduced KIR expression^41–43^. Beside a transition from precursors, the role of NKG2A we identified in maintaining NK cell expansion capacity could help explain the overrepresentation of NKG2A^+^ over NKG2A^−^ NK cell populations with an expansion advantage mediated by NKG2A in the post-HCT setting rich in cytokines that provide stimulatory signaling^44,45^. Likewise, NKG2A^+^ NK cells are preferentially found in the myeloid cell-rich^46,47^ tumor microenvironment of human cancers^48–50^, where functionally, intratumoral NK cells are generally described as hyporesponsive^51^.

While our work identified a key role for NKG2A in maintaining NK cell expansion, although not directly targeted, we also found that expression of KIRs is essential for expansion. Indeed, NKG2A^−^KIR^−^ NK cells displayed the lowest expansion capacity although not being depleted of the immature CD56^bright^CD16^−^ NK cell subset^52^, but having higher expression of the inhibitory receptor LILRB1 instead, that like NKG2A and KIR also interacts with HLA class I molecules^53^. Thus, studying the impact of KIR and LILRB1 on regulating NK cell expansion is warranted. Together, it is possible that inhibitory signaling is required more generally to avoid activation-induced cell death and thus sustain expansion of NK cells confronted with excessive stimulatory signals. Ultimately, this may represent part of the mechanisms for maintaining self-tolerance, by which the expansion of NK cells lacking essential inhibitory receptors like NKG2A for self-HLA class I is reduced to protect against autoimmunity and tissue damage in inflammatory settings. On the other hand, cancer may exploit this mechanism to evade NK cell mediated immune surveillance by upregulation of HLA-E^9–13,40^, leading to preferential expansion of NKG2A^+^ NK cells and thus depletion of potentially more responsive, uninhibited NK cells lacking NKG2A. This should be considered in cancer immunotherapy approaches targeting NKG2A on NK cells because this could drive activation-induced cell death leading to reduced survival of these cells, if not counter-balanced by signaling from inhibitory receptors like KIRs.

## Supplementary Information

Supplementary Figures.

## Data Availability

The data generated in this study are available upon request from the corresponding author.
