# Supplementary material for: The NK cell checkpoint NKG2A maintains expansion capacity of human NK cells
Source: Sci Rep. 2023 Jun 29;13:10555. doi: 10.1038/s41598-023-37779-6 (PMC10310841; doi:10.1038/s41598-023-37779-6)
Supplement: Supplementary file 1 — Supplementary Figures. [file 41598_2023_37779_MOESM1_ESM.pdf]

SUPPLEMENT

Supplementary Figures 1 - 8

Kaulfuss et al.

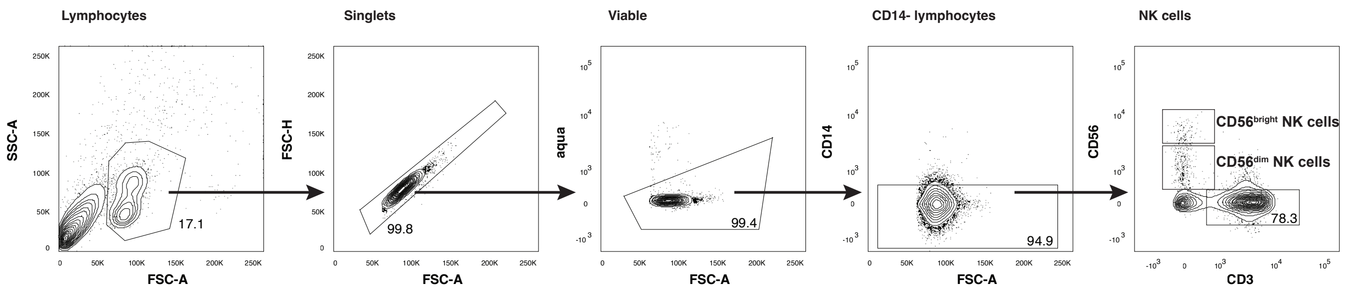

Supplementary Figure 1

Supplementary Figure 1

Gating strategy for phenotyping of $CD3^{-}CD56^{+}$ NK cells.

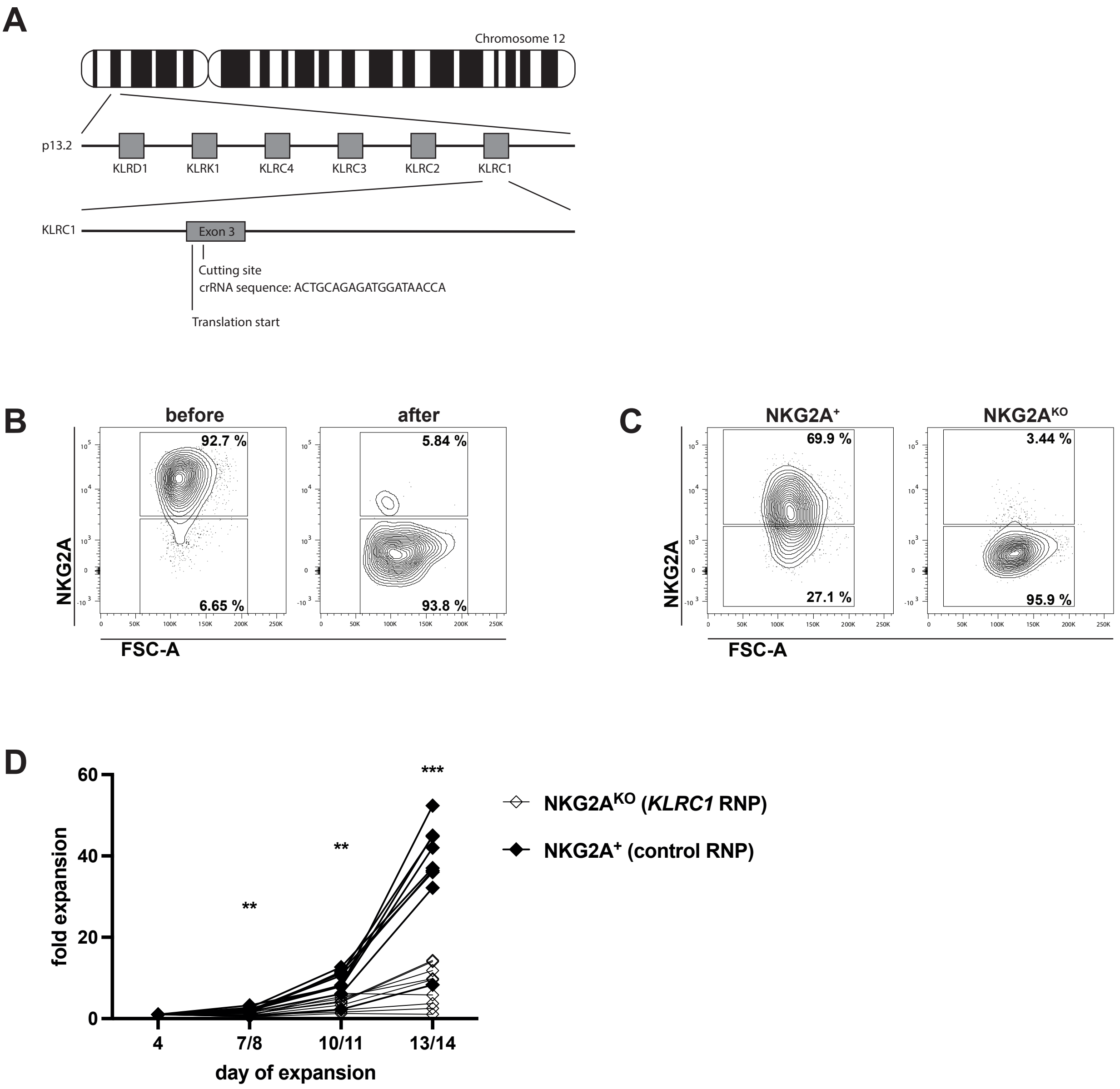

Supplementary Figure 2

Supplementary Figure 2

(**A**) A crRNA targeting exon 3 of *KLRC1* located on chromosome 12. (**B**) Representative FACS plots of NKG2A expression on $CD3^{-}CD56^{+}$ NK cells before (left) and after (right) knockout of NKG2A. (**C**) Representative FACS plots of NKG2A expression on $NKG2A^{+}$ (left) or $NKG2A^{KO}$ (right) NK cells after expansion. (**D**) $NKG2A^{+}$ NK cells were FACS sorted and either electroporated with Cas9 RNP targeting *KLRC1* ($NKG2A^{KO}$) or nontargeting control Cas9 RNP ($NKG2A^{+}$) and expanded on K562mbIL21 feeder cells as described in the methods section. Symbols represent single donors; 9 donors. *P* values were calculated using 2way ANOVA with Šídák's multiple comparisons test.

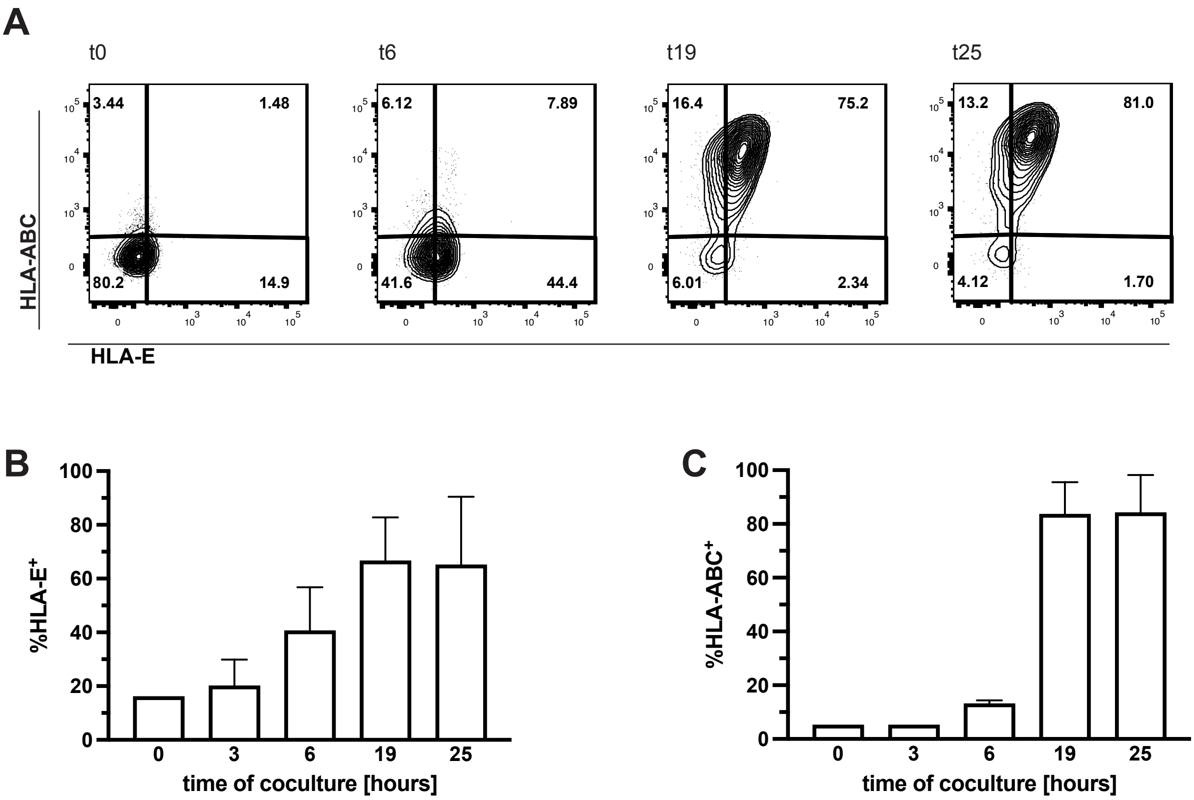

Supplementary Figure 3

Supplementary Figure 3

(**A**) Representative FACS plots of HLA-E and HLA class I (HLA-ABC) expression on PKH26 labeled K562mbIL21 feeder cells upon coculture with human NK cells. NK cells were isolated with a negative NK cell isolation kit by MACS from PBMCs of 2 donors and co-cultured with K562mbIL21 feeder cells as per expansion protocol described in the methods section. (**B**) Quantification of HLA-E expression on K562mbIL21 feeder cells at indicated time points of coculture with NK cells. **(C**) Quantification of HLA class I (HLA-ABC) expression on K562mbIL21 feeder cells at indicated time points with NK cells.

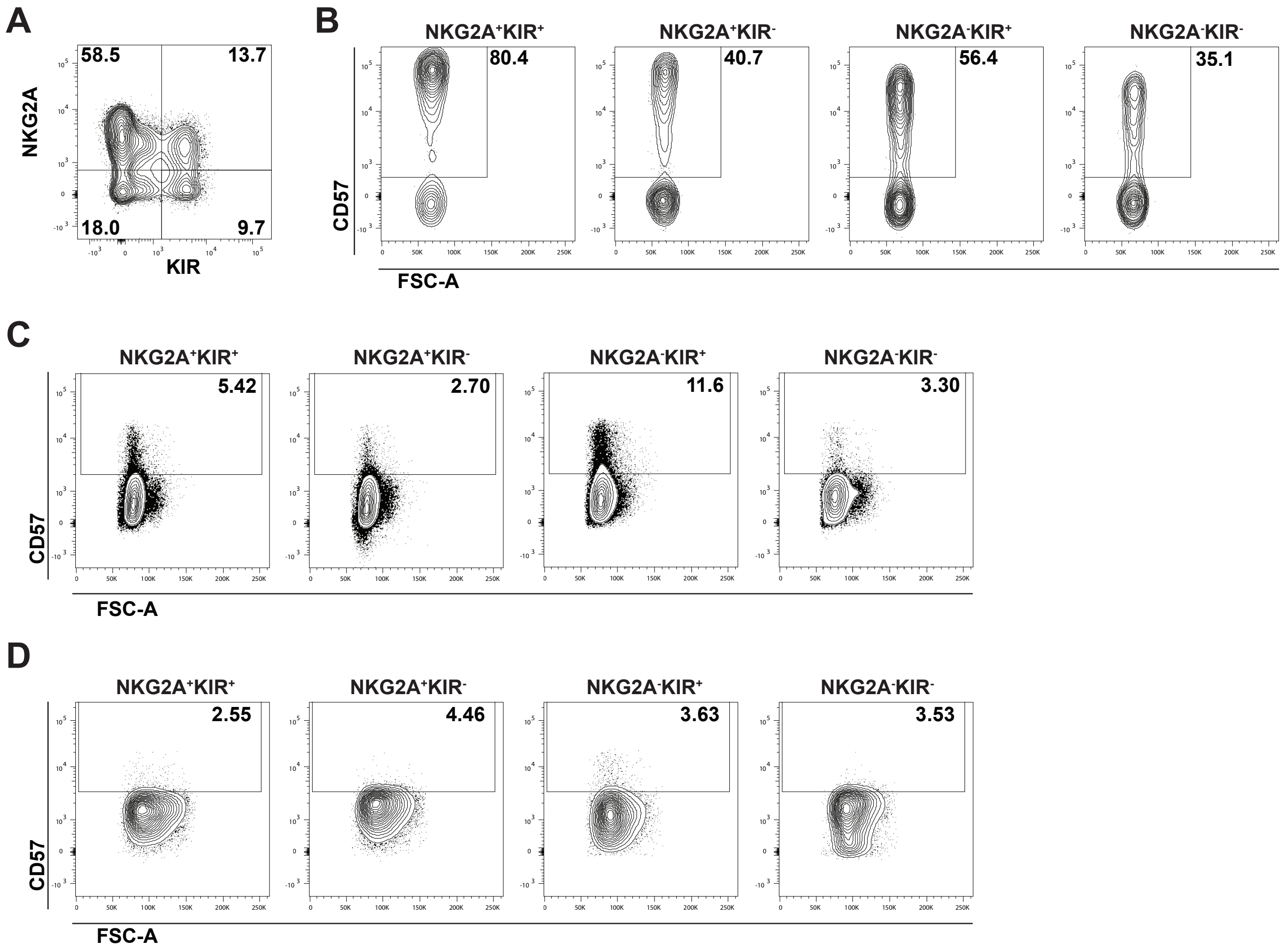

Supplementary Figure 4

Supplementary Figure 4

(**A**) Representative FACS plot of the expression of NKG2A and KIR on $CD3^-CD56^+$ NK cells before expansion. (B-D) Representative FACS plot of the expression of CD57 on the four different subsets of $CD3^-CD56^+$ NK cells before expansion (**B**) on day 13 (**C**) and on day 20 (**D**) after sort.

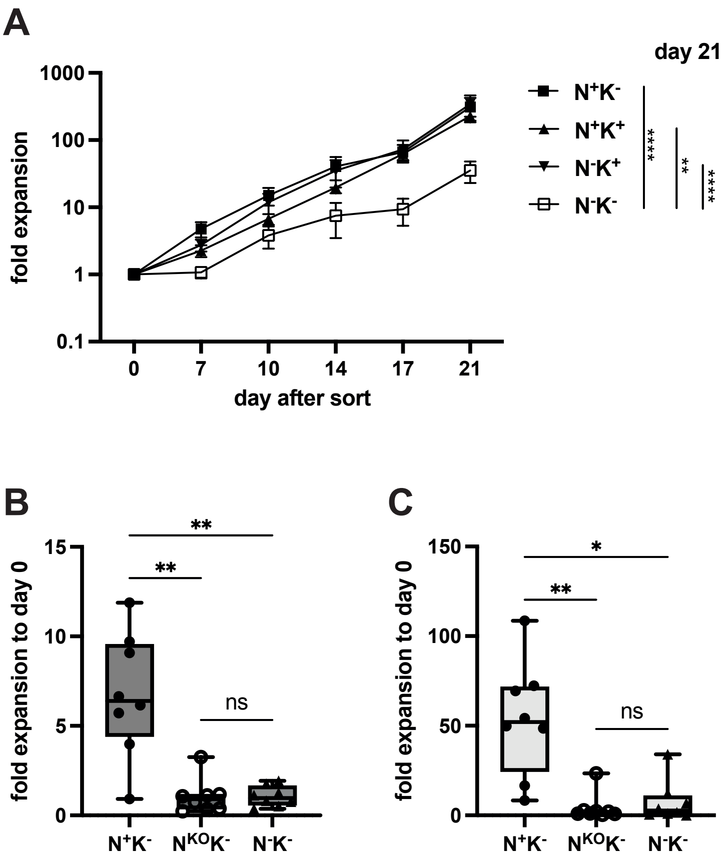

Supplementary Figure 5

Supplementary Figure 5

(**A**) Expansion of NKG2A$^+$KIR$^+$ (N$^+$K$^+$), NKG2A$^+$KIR$^-$ (N$^+$K$^-$), NKG2A$^-$KIR$^+$ (N$^-$K$^+$) and NKG2A$^-$KIR$^-$ (N$^-$K$^-$) NK cell subsets based on sorted phenotype (**p=0.0014 for N$^+$K$^+$ vs N$^-$K$^-$; ****p<0.0001 for N$^+$K$^-$ vs N$^-$K$^-$; ****p<0.0001 for N$^-$K$^+$ vs N$^-$K$^-$). (B, C) Expansion of NKG2A$^+$KIR$^-$ (N$^+$K$^-$), NKG2A^{KO}KIR$^-$ (N^{KO}K$^-$) and NKG2A$^-$KIR$^-$ (N$^-$K$^-$) NK cell subsets based on sorted phenotype at day 7 (**B**, **p=0.0059 for N$^+$K$^-$ vs N^{KO}K$^-$; **p=0.0045 for N$^+$K$^-$ vs N$^-$K$^-$) and day 14 (**C**, **p=0.0066 for N$^+$K$^-$ vs N^{KO}K$^-$; *p=0.016 for N$^+$K$^-$ vs N$^-$K$^-$) after knockout. Mean values ± SEM are shown, symbols represent single donors; 3-8 donors. *P* values were calculated using 2way ANOVA with Bonferroni's multiple comparison (A) and 1way ANOVA with Bonferroni's multiple comparison (B, C).

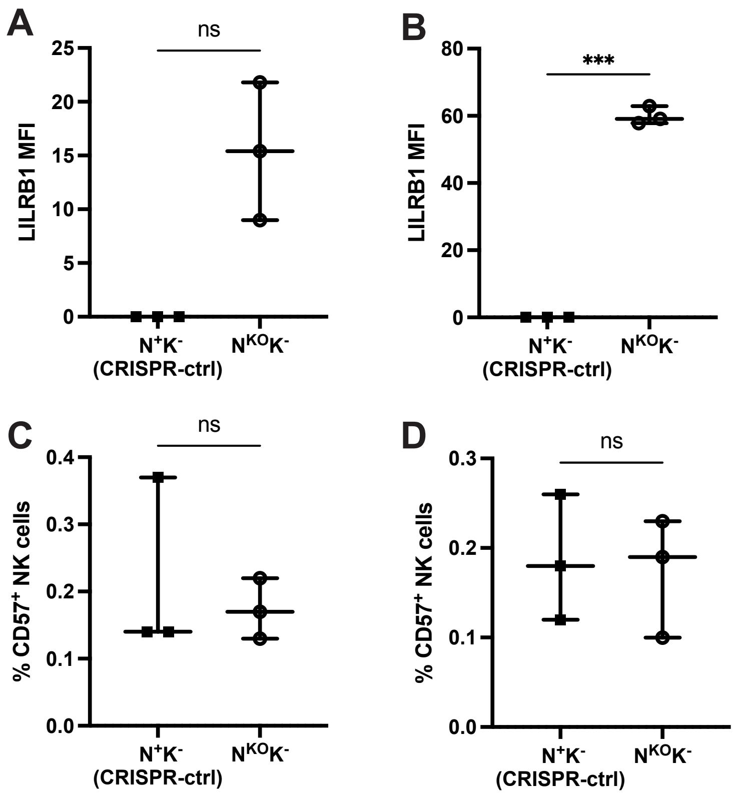

Supplementary Figure 6

Supplementary Figure 6

(A, B) Intensity of LILRB1 on expanding mock-edited NKG2A^{+}KIR^{-} NK cells (CRISPR-ctrl N^{+}K^{-}) and NKG2A^{KO}KIR^{-} NK cells (N^{KO}K^{-}) on day 6 (**A**) and on day 13 (**B**, ***p=0.0007) after knockout. (C, D) Expression of CD57 on the subsets on day 6 (**C**) and on day 13 (**D**) after knockout. Mean values ± SEM are shown, symbols represent single donors; 3 donors. *P* values were calculated using paired t-test.

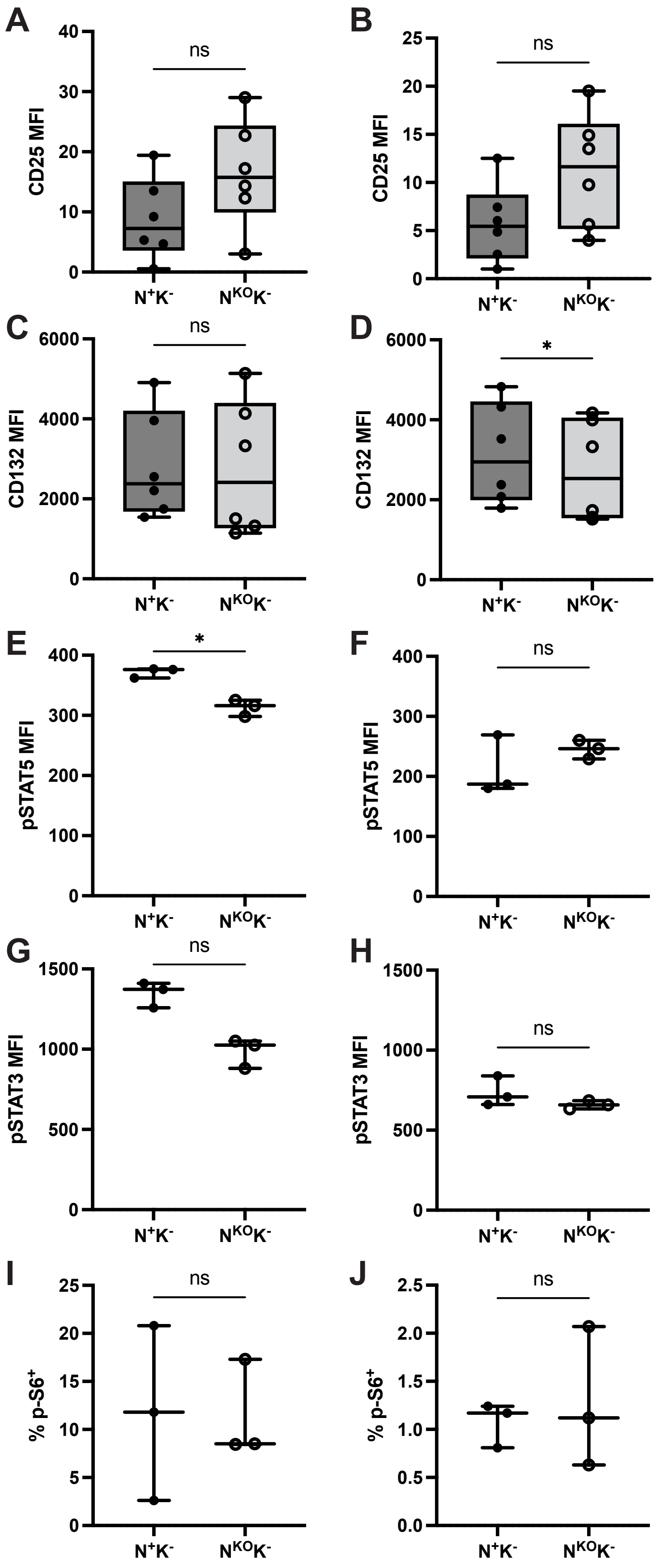

Supplementary Figure 7

Supplementary Figure 7

(A, B) Intensity of IL-2Rα (CD25) on $NKG2A^{+}KIR^{-}$ ($N^{+}K^{-}$) NK cells and $NKG2A^{KO}KIR^{-}$ ($N^{KO}K^{-}$) NK cells on day 6 (**A**) and on day 13 (**B**) after knockout. (C, D) Intensity of IL-2Rγ (CD132) on $NKG2A^{+}KIR^{-}$ NK cells and $NKG2A^{KO}KIR^{-}$ NK cells on day 6 (**C**) and on day 13 (**D**, *p=0.0293) after knockout. (E, F) Intensity of phosphorylated STAT5 (pSTAT5) on $NKG2A^{+}KIR^{-}$ NK cells and $NKG2A^{KO}KIR^{-}$ NK cells on day 6 (**E**, *p=0.0387) and on day 13 (**F**) after knockout. (G, H) Intensity of phosphorylated STAT3 (pSTAT3) on $NKG2A^{+}KIR^{-}$ NK cells and $NKG2A^{KO}KIR^{-}$ NK cells on day 6 (**G**) and on day 13 (**H**) after knockout. (I, J) Expression of pS6 on day 6 (**I**) and on day 13 (**J**) after knockout. Mean values ± SEM are shown, symbols represent single donors; 3-6 donors. *P* values were calculated using paired t-test.

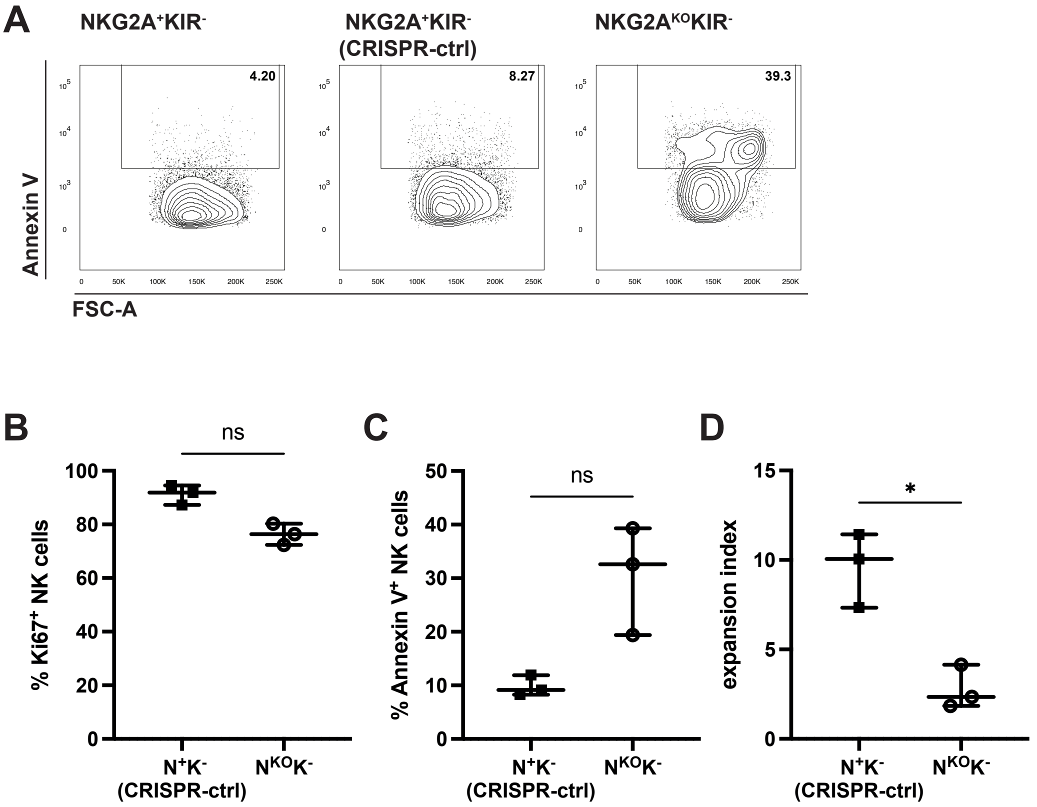

Supplementary Figure 8

Supplementary Figure 8

(**A**) Representative FACS plots of the binding of Annexin V to viable NKG2A^{+}KIR^{-} NK cells, mock-edited NKG2A^{+}KIR^{-} (CRISPR-ctrl N^{+}K^{-}) NK cells or NKG2A^{KO}KIR^{-} (N^{KO}K^{-}) NK cells on day 6 after knockout. (B-D) Expression of Ki-67 in mock-edited NKG2A^{+}KIR^{-} NK cells and NKG2A^{KO}KIR^{-} NK cells (**B**), binding of Annexin V to mock-edited NKG2A^{+}KIR^{-} NK cells and NKG2A^{KO}KIR^{-} NK cells (**C**) and expansion index in mock-edited NKG2A^{+}KIR^{-} NK cells and NKG2A^{KO}KIR^{-} NK cells (**D**, *p=0.0397) on day 6 after knockout. Mean values ± SEM are shown, symbols represent single donors; 3 donors. *P* values were calculated using paired t-test.
